# Technical efficiency of district hospitals: Evidence from Namibia using Data Envelopment Analysis

**DOI:** 10.1186/1478-7547-4-5

**Published:** 2006-03-27

**Authors:** Eyob Zere, Thomas Mbeeli, Kalumbi Shangula, Custodia Mandlhate, Kautoo Mutirua, Ben Tjivambi, William Kapenambili

**Affiliations:** 1 World Health Organization, P.O. Box 30309, Lilongwe, Malawi; 2 Ministry of Health and Social Services, Private Bag 13198, Windhoek, Namibia; 3 World Health Organization, P.O. Box 3444, Windhoek, Namibia

## Abstract

**Background:**

In most countries of the sub-Saharan Africa, health care needs have been increasing due to emerging and re-emerging health problems. However, the supply of health care resources to address the problems has been continuously declining, thus jeopardizing the progress towards achieving the health-related Millennium Development Goals. Namibia is no exception to this. It is therefore necessary to quantify the level of technical inefficiency in the countries so as to alert policy makers of the potential resource gains to the health system if the hospitals that absorb a lion's share of the available resources are technically efficient.

**Method:**

All public sector hospitals (N = 30) were included in the study. Hospital capacity utilization ratios and the data envelopment analysis (DEA) technique were used to assess technical efficiency. The DEA model used three inputs and two outputs. Data for four financial years (1997/98 to 2000/2001) was used for the analysis. To test for the robustness of the DEA technical efficiency scores the Jackknife analysis was used.

**Results:**

The findings suggest the presence of substantial degree of pure technical and scale inefficiency. The average technical efficiency level during the given period was less than 75%. Less than half of the hospitals included in the study were located on the technically efficient frontier. Increasing returns to scale is observed to be the predominant form of scale inefficiency.

**Conclusion:**

It is concluded that the existing level of pure technical and scale inefficiency of the district hospitals is considerably high and may negatively affect the government's initiatives to improve access to quality health care and scaling up of interventions that are necessary to achieve the health-related Millennium Development Goals. It is recommended that the inefficient hospitals learn from their efficient peers identified by the DEA model so as to improve the overall performance of the health system.

## Background

At the Millennium Summit in 2000, Member States of the United Nations (UN) reaffirmed their commitment to eradicate world poverty and improve the health and welfare of the world's poorest by 2015 [1]. Health is at the centre of the MDGs. Three of the eight goals are health MDGs – MDGs 4, 5 and 6 related to child health, maternal mortality and diseases such HIV, tuberculosis and malaria respectively. Besides, health contributes significantly to the achievement of the other MDGs [1].

The achievement of the health-related Millennium Development Goals (MDGs) and related initiatives, among other things, requires the availability of adequate resources for the health sector to improve access and quality of care. However, given the poor macro-economic performance of most countries in the Africa region, the resources required to meet the costs of achieving the development goals are far beyond the reach of many.

In sub-Saharan Africa, hospitals absorb the greatest proportion of the total health expenditure, which is estimated at 45–69% of government health sector expenditure [2,3]. Namibia is no exception to this. Thus, the technical efficiency of hospitals merits close scrutiny in order to optimize the utilization of the available health care resources and mobilize additional resources for the health system through efficiency savings.

Evidence emerging from various studies indicates the wide prevalence of technical inefficiency of hospitals as well as other health facilities in Africa [4,5]. With high levels of technical inefficiency, a significant proportion of the available resources are wasted. This further compounds the existing shortage of resources experienced by many countries in the region.

To date, no studies of technical efficiency have been conducted in Namibia using frontier techniques of efficiency measurement. Hence, it is vital to assess the technical efficiency of district hospitals using more robust measures of efficiency measurement in order to be able to utilize the available resources optimally and expedite the move towards achieving health and development goals.

The objective of this paper is therefore to examine the technical efficiency of district hospitals in Namibia with a view to assess the *status quo *in productive efficiency and quantify the possible efficiency gains that can be ploughed back into the system and bridge the resource gap currently existing.

## Brief country profile

Namibia is located in the South-western part of the African continent and has a surface area of 824,116 square kilometres. The country is divided into 13 administrative regions. The demographic, socio-economic and epidemiological profile of the country is depicted in Table 1.

**Table 1:** Namibia – Health and development indicators

Characteristic	Value
Total population (2001)	1,830,330
Annual population growth rate (%)	2.6
Life expectancy at birth (male/female) (years)	48/50
Infant mortality rate (per 1000 live births)	38
Under-five mortality rate (per 1000 live births)	62
Total fertility rate	4.2
Maternal mortality ratio (per 100,000 live births)	271
Stunting in under-five children (%)	24.0
Gross national income per capita (2002) (US$)	1,780
Population living below US$1 a day (%)	35
Gini coefficient	0.70
Human development index, 2004	0.607
HIV prevalence rate (%)	19.8
Prevalence of tuberculosis (per 100,000)	635
Malaria mortality rate (per 100,000)	39
Per capita total expenditure on health (international dollars) (2001)	342

Sources: [6-12]

Communicable diseases account for the greatest proportion of the disease burden. However, as concomitants of the demographic and epidemiological transition, non-communicable diseases are also on the increase.

The country's health policy is based on the tenets of the Primary Health Care strategy, which include equity, community involvement, multi-sectoral collaboration and appropriate technology. There are 13 Regional Health Management Teams that oversee service delivery in 34 health districts.

The provision of health services in Namibia is split between three main providers – Government (70–75%), missions (15–20%), and the private sector (5%). The missions (Lutheran, Roman Catholic and Anglican) are not-for-profit providers, and predominantly work in rural areas.

Pre-independence, mission facilities were providing health services to the Namibian population mainly in the northern and to a lesser degree in the southern parts of the country. After independence, government entered into an agreement with the mission health facilities for them to continue to provide services in the areas that they have been operating and for government not to construct health facilities in the same areas to avoid duplication. They are 100% subsidized by the Ministry of Health and Social Services (MOHSS). Personnel are remunerated according to Government rates.

The for-profit private sector is mainly urban-based, providing health care from eleven medium-sized private hospitals, private pharmacies, doctors' surgeries and nursing homes.

The MOHSS has adopted a decentralization policy to improve service provision and management by de-concentrating authority to 13 MoHSS Regional Directorates. At the national level re-organization has been undertaken to enable the national level to support service provision and management development for the whole health sector. The 13 Regional Directorates oversee service delivery in a total of 34 health districts.

There are 30 public district hospitals providing institutional medical and nursing care, including preventive, promotive and curative health care. They also provide technical and referral support to 37 health centres and 259 clinics. Furthermore, there are three intermediate and one national referral hospitals that function as referral centres for the district hospitals.

The total health expenditure (THE) per capita in Namibia in 2001 was US$ 154. This constitutes about 6% of the GDP. Furthermore the government allocates a little more than 12% of its budget for health care [13]. THE per capita in Namibia compares very favorably to those in most countries in sub-Saharan Africa, which are far from the US$ 34 recommended by the WHO Commission on Macroeconomics and Health to provide a basic package of services [14]. The government is the main financier of health (more than 80%). The contribution of donors and households as sources of health finance is relatively small.

## Techniques of hospital efficiency measurement

The measurement of efficiency in healthcare is a difficult exercise for various reasons including the complex nature of the productive process and difficulty in measuring the ideal output of the sector, i.e. improved health status.

Technical efficiency attempts to address two questions depending on whether it has input- or output-orientation. In output-oriented technical efficiency the focus is on expanding output quantities without changing the quantity of inputs used. On the other hand, input-oriented technical efficiency focuses on reducing input quantities used without changing the quantity of outputs produced.

Inappropriate size of a hospital (too large or too small) may sometimes be a cause for technical inefficiency. This is referred to as scale inefficiency and takes two forms – decreasing returns to scale and increasing returns to scale. Decreasing returns to scale (also known as diseconomies of scale) implies that a hospital is too large for the volume of activities that it conducts. Unit costs increase as outputs increases. In contrast, a hospital with increasing returns to scale (economies of scale) is too small for its scale of operation. Unit costs decrease as outputs increase. A hospital that is scale-efficient is said to operate under constant returns to scale.

The performance of hospitals may be measured using ratios that mainly measure capacity utilization and frontier techniques founded on micro-economic theory of production. Commonly used ratios include: bed occupancy rate, turnover ratio, turnover interval and average length of stay. Frontier methods of efficiency measurement include linear programming techniques (e.g. data envelopment analysis) and econometric techniques (e.g. production and cost functions). The current study employs data envelopment analysis, which is briefly described in the following section.

### Data envelopment analysis (DEA)

DEA was first introduced by Charness *et al *in 1978 for measuring the relative efficiency of organizations such as hospitals and schools that lack the profit maximization motive [15]. DEA uses linear programming techniques to compute the efficiency scores for each hospital. Hospitals that are technically efficient have a score of 1 or 100%, whereas inefficient hospitals have efficiency scores of less than 1 (i.e. less than 100%). Constant returns to scale DEA linear programming model is depicted here under.



Subject to:



Where:

*y*_*rj *_= amount of output *r *from hospital *j*

*x*_*ij *_= amount of input *i *to hospital *j*

*u*_*r *_= weight given to output *r*

*v*_*i *_= weight given to input *i*

*n *= number of hospitals

*s *= number of outputs

*m *= number of inputs

In DEA the efficiency of an organization (district hospitals in this case) is measured relative to a group's observed best practice. This implies that the benchmark against which to compare the efficiency of a particular district hospital is determined by the group of district hospitals in the study and not a value fixed by hospitals outside of the group.

The basic DEA model helps to find answers to questions such as:

(i) Which district hospitals (or hospital departments) are the most efficient?

(ii) If all district hospitals are to perform according to best practice (i.e. the efficient peer hospitals), by how much could inputs/resources be reduced to produce the current output levels; or alternatively, by how much could outputs be increased with the current input levels?

(iii) How much resources can be potentially saved if all district hospitals are operating at an optimal scale?

(iv) Which of the efficient district hospitals can serve as role models for the inefficient ones (so that their method of doing business may be emulated)?

DEA easily accommodates multiple inputs and outputs without the requirement for a common denominator of measurement. This makes it particularly suitable for analyzing the efficiency of hospitals as they use multiple inputs to produce many outputs. Furthermore, it provides specific input and output targets that would make an inefficient hospital relatively efficient. It also identifies efficient peers for those hospitals that are not efficient. This helps the inefficient hospitals to emulate the functional organization of their peers so as to improve their efficiency.

However, like many other empirical methods, DEA has its limitations. First, it produces results that are sensitive to measurement error. For example, if one hospital's inputs are understated or its outputs overstated, it can become an outlier and significantly reduce the efficiency of other hospitals. Second, DEA measures efficiency relative to the best practice within hospitals in the particular sample. Therefore, it is not possible to compare how district hospitals in Namibia fare relative to their counterparts in South Africa or Zimbabwe with respect to technical efficiency [16].

## Data and methods

### Sampling

The study focuses on the entire population of district hospitals in Namibia (N = 30) including both public sector and mission hospitals. The hospitals are distributed over the 13 regions of the country.

### Selection of inputs and outputs

Improved health status is the ultimate output of hospitals or the health system at large. However, due to difficulties in accurately measuring improvements in health status, hospital output is measured by an array of intermediate health services that supposedly improve health status [17]. Due to data constraints, the empirical DEA model is based on three inputs (total recurrent expenditure, beds and nursing staff) and two outputs (total outpatient visits and inpatient days).

Recurrent expenditure covers salaries and benefits, pharmaceuticals, supplies, equipment and services such as catering. Although the recurrent expenditure includes salaries of the nursing staff, the aggregate recurrent expenditure does not give a very clear picture about the type of staff in the health facilities. It is affected by the staff mix. A hospital with a greatest proportion of higher-level cadres will definitely have a higher salary bill even if the number of staff is small. Disaggregating the salary component by health worker type was difficult due to weak health information system in the facilities. As nurses constitute the greatest proportion of the health workforce, the number of the nursing staff was entered as an input in order to increase the policy relevance of the findings.

It is assumed that the effect of inflation is not a problem to influence the findings, as the rate of inflation is uniform among the hospitals. Salary scales are uniform and drugs and supplies are procured centrally. Even if one would use a deflated price, it will always be the same as we are using the same rate throughout the regions.

The selection of inputs and outputs for a DEA study requires a careful thought as the distribution of efficiency is likely to be affected by the definition of outputs and the number of inputs and outputs included [18]. Two schools of thought dominate the discussion on the definition and measurement of the output of health care organisations [19]:

i. the *process approach*, which asserts that the output of a health care organisation consists of services provided by the different units such as the X-rays, laboratory procedures, patient days *etc*; and

ii. the *outcomes approach*, regards the above processes only as intermediate steps leading to the desired change in patient's health status. According to this approach, therefore, output should be measured in terms of the end result or outcome, that is improved health.

Although there is a general consensus that the ultimate measure of output should be an improvement in the quantity and quality of life, practical difficulties limit the use of the outcomes approach [19]. First, it is easier to measure and define processes (services) in health care than changes in health status. Second, changes in health outcome can not be entirely attributed to health care. Health is multi-dimensional and affected significantly by a host of other socio-economic factors. Consequently, output is measured as an array of intermediate outputs (health services) that supposedly improve health status [17].

Buttler [20] classifies hospital output into four broad categories: inpatient treatment, outpatient treatment, teaching and research. Measuring hospital output by such variables as inpatient days or outpatient visits, does not capture the case-mix and the quality of service rendered. Eventhough the use of Diagnosis-related groups (DRGs) may handle the problem of hospital case-mix, the absence of data makes its use limited in most developing countries. Within the context of developing countries, stratifying hospitals according to their level may to some degree take account of the case-mix and factors such as staffing pattern and medical technology used that are likely to affect the quality of care delivered.

Inputs in hospital production are classified as labour, capital and supplies. The labour input can be disaggregated into the various professional groups such as physician, nurse and administrative staff. In most studies, capital is proxied by the number of hospital beds.

Thus, in the present study, two hospital outputs are identified for the DEA model: outpatient visits and inpatient days. These are the major outputs of the district hospitals under consideration, as their involvement in teaching and research is very minimal or non-existent.

### Data collection

Data was collected using a questionnaire that included information on inputs, outputs. The period covered includes the financial years 1997/98 to 2000/2001.

### Data analysis

The technical efficiency scores are computed using data envelopment analysis programme, version 2.1 (DEAP 2.1) designed by Coelli [21]. Hospital utilization ratios are also computed using Microsoft Excel.

Input-oriented model was used in this study, as we think that the decision to use or not to use district hospital services is at the discretion of the consumer/client/patient. It is an exogenous factor that hospital managers may not have total control of.

To test for the robustness of the DEA technical efficiency scores, the Jackknife analysis was used. In the jackknife analysis, a limited number of samples are obtained by omitting one observation at a time [22]. In this case the efficient hospitals are dropped one at a time from the analysis and the efficiency scores re-estimated. The similarity of the efficiency rankings between the model with all the hospitals included and those based on dropping each of the efficient hospitals is then tested by using Spearman rank correlation coefficient. A correlation coefficient of 1 implies that the rankings are exactly the same. A value of zero indicates the absence of correlation between the rankings and reverse ranking is implied by a value of -1.

## Results

### General description

Data was complete in the required variables for only 26 hospitals. The findings indicate a wide variation in the size of the district hospitals as indicated by the authorized number of beds. Summary statistics of the key variables is given in Table 2.

**Table 2:** Summary Statistics

Variable	Mean	Standard deviation	Minimum	Maximum
FY 1997/1998				

Recurrent expenditure (N$)*	7,158,567	4,379,763	1,041,142	20,505,989
Beds (authorized)	128	79	40	450
Nursing staff	66	47	26	262
Outpatient visits	32653	39553	2151	135696
Inpatient days	26158	19367	4628	92745

FY 1998/1999				

Recurrent expenditure (N$)	8,277,652	5,191,196	2,673,900	22,266973
Beds (authorized)	129	79	40	450
Nursing staff	67	48	26	273
Outpatient visits	38772	39034	1996	139293
Inpatient days	31762	24331	5230	119126

1999/2000				

Recurrent expenditure (N$)	9,392,129	5,778,991	3,416,603	29,754,220
Beds (authorized)	130	78	40	450
Nursing staff	68	49	26	272
Outpatient visits	38110	42155	809	172498
Inpatient days	30851	24941	5537	125278

2000/2001				

Recurrent expenditure (N$)	12,038,075	8,304,679	4,557,855	45,227,355
Beds (authorized)	130	78	40	450
Nursing staff	68	48	22	261
Outpatient visits	30,780	27,501	2,104	97,998

Inpatient days	37,372	36,875	8,579	183,654

* The average US$ – N$ exchange rate was 5.53, 6.27, 6.95 and 8.67 over the four years period.

### Capacity utilization measures

There is a wide variation among the district hospitals in terms of capacity utilization as measured by bed occupancy rate, bed turnover ratio and average length of stay. Table 3 depicts this information for the years included in the study, 1997/1998 to 2000/2001 Financial Year.

**Table 3:** Capacity utilization measures, 1997/98–2000/2001

Indicator	Mean	SD	Minimum	Maximum
**1997/1998**				

Bed occupancy rate (%)	55	19	17	98
Bed turnover ratio	32	13	9	73
Average length of stay	7	2	2	10

**1998/1999**				

Bed occupancy rate (%)	58	21	10	103
Bed turnover ratio	33	15	7	87
Average length of stay	7	2	2	12

**1999/2000**				

Bed occupancy rate (%)	57	23	8	107
Bed turnover ratio	33	13	9	64
Average length of stay	6	2	2	12

**2000/2001**				

Bed occupancy rate (%)	67	28	18	135
Bed turnover ratio	38	15	9	73
Average length of stay	7	2	3	12

It is observed that the mean occupancy rates for all the years are much less than the conventionally accepted levels of 80–85% occupancy rate. Furthermore, some of the hospitals have occupancy rates that are very low even compared to the means of the district hospitals included in the study.

### Technical efficiency scores from DEA model

The constant returns to scale (CRS) DEA models estimated for the period 1997/98 to 2000/2001 indicate average technical efficiency scores ranging from 62.7% to 74.3%. The jackknife analysis indicates that the stability of the estimates and that the efficiency frontier has not been affected by extreme outliers (Spearman rank correlation coefficient = 0.99). A summary of the technical efficiency scores is given in Table 4.

**Table 4:** DEA technical efficiency (TE) scores from VRS model, 1997/98–2000/2001

	Efficiency score (%)							
	
	1997/98		1998/99		1999/2000		2000/2001	
	
Hospital	Technical	Scale	Technical	Scale	Technical	Scale	Technical	Scale
Andara	90.3	99.6	78.6	88.6	71.6	71.6	100	100
Eenhana	100	100	100	100	100	100	100	100
Engela	98.6	98.6	100	100	100	100	100	100
Gobabis	100	100	100	100	97.1	97.1	83.3	96.4
Grootfontein	32.9	76.5	40	87.8	33	75.2	28.9	65.3
Karasburg	100	100	100	100	74.4	74.4	57.2	57.2
Katima Mulilo	100	100	100	100	100	100	77.6	95.8
Keetmanshoop	45.6	100	43.7	100	33.8	76.3	39.6	80.6
Kongo	73.1	79.2	72.4	75.2	66.3	66.3	52.6	52.6
Luderitz	76.5	86.3	77.3	79.7	65.9	69.3	100	100
Nankudu	74.4	74.4			39.4	44.5	46.3	53.5
Nyangana	100	100	93	93	78	82.7	76	85.9
Okahandja	41.6	41.6	48.9	48.9	51.5	51.5	44.7	44.7
Okahao	25.7	39.6	75.7	78.9	66.7	85.1	22	36.8
Okakarara	52.3	65.8	48.3	56.7	28	29.7	37	41.4
Omaruru			100	100	50.6	63.7	69.6	81.5
Onandjokwe	60.3	60.3	74.7	74.7	62.4	62.4	89.7	89.7
Oshikuku			98.3	98.3	53.9	94.2	80.8	98
Otjiwarongo			77.5	87.6	71.2	77	68.1	83.2
Outapi	100	100	100	100	95.7	95.7	100	100
Rehoboth	63.3 99.9			45	78.1	60.8	83.7	
Swakopmund	48.5	85.4	49.2	74.3	44.3	75.1	41.5	70.6
Tsandi					64.1	69.3	94.8	94.8
Tsumeb			34.9	57.3	40.2	61	73.7	77.5
Usakos	11.9	15.3	7.3	7.3	6.2	7.8	5.7	7.5
Walvis Bay	100	100	100	100	90.9	96.2	90	99
**Mean**	**71.6**	**81.1**	**74.3**	**83.7**	**62.7**	**73.2**	**66.9**	**76.8**

NB: shaded areas imply that data was not available

The CRS TE scores in the above table indicate that throughout the period considered, less than half of the district hospitals were located on the frontier (TE score = 100%). Furthermore, it is revealed that there are hospitals whose TE scores are extremely low.

The CRS technical efficiency scores reveal combined inefficiency that is due to both pure technical inefficiency and inefficiency that is due to inappropriate hospital size. The table further reveals that scale inefficiency is as equally prevalent as pure technical inefficiency. Increasing returns to scale is the predominant form of scale inefficiency observed.

### Input savings

Inefficiency levels ranging from 26–37% are observed. This implies that if the inefficient hospitals were to operate as efficient as their peers on the best-practice frontier, the health system could have reaped efficiency gains amounting to 26–37 % of the total resources used in running the hospitals. The possible input savings are depicted in Table 5 below.

**Table 5:** Input savings from district hospitals, 1997/98–2000/2001

	Input savings in the Financial Year:			
	
Input type	1997/98	1998/99	1999/00	2000/01
Recurrent expenditure (N$)	27,009,153	43,277,100	66,151,450	73,985,129
Bed	309	411	633	632
Nursing staff	190	212	443	348

The above-mentioned input savings are aggregates for the whole system. The amounts of input savings for each hospital are given elsewhere [23].

### Best-practice hospitals

In DEA, the frontier against which the technical efficiency of all hospitals is measured is defined by those hospitals in the group with a TE score of 100%. The hospitals producing on the efficient frontier define the best practice and thus could be regarded as role models. For each inefficient hospital the DEA model has identified efficient hospitals that could be used as comparators. The inefficient hospitals are expected to learn from their efficient peers by observing their production process.

## Discussion, conclusion and recommendations

The results of this study indicate that many of the district hospitals operate at technical efficiency levels well below the efficient frontier. The findings of this study are in line with other studies in sub-Saharan Africa, which indicate the wide prevalence of technical inefficiency [e.g. [4,24]]. For example, Zere et al. [4], in their study of technical efficiency and productivity of public sector hospitals in South Africa found technical inefficiency levels ranging between 34% – 48%.

The inefficiency levels observed suggest a substantial amount of input savings, which could go a long way in injecting additional resources to the health system to address the backlog of inequities and/or further improve the quality of the available health care. For example, the efficiency saving that could have been realized in 2000/2001 is equivalent to the amount needed for the construction of 50 clinics.

The study further reveals that the prevalent scale inefficiency is increasing returns to scale. In the presence of increasing returns to scale, expansion of outputs reduces unit costs. However, increasing the level of outputs requires an increase in the demand for health care, which is beyond the control of the hospital management. Merger of hospitals in close proximity to one another may be an option worth of consideration. However, this option may potentially pose some problems given the very low population density of the country (2 persons per square kilometre). If larger hospitals are to be established in centrally located places, residents of some areas may incur additional costs in travel expenditure and in delayed treatment of emergency cases [4]. These potential problems may to some extent be minimized by establishing primary care units linked to centrally located hospitals through an effective referral and patient transport system. In taking such decisions, however, the equity implications should always be viewed carefully.

Finally, given the immense task of redressing past inequities on the one hand, and the relatively dwindling health care resources owing to increased needs, it is important that efficiency measures be instituted and pursued vigorously to contribute to improvements of the health status of the population.

## Limitation of the study

In the presence of good panel data for a sufficiently longer period of time it is important to estimate DEA-based Malmquist productivity index (MPI) to observe the changes in efficiency and those changes in productivity that are accounted for by technological change. However, this was not done, as the data that we have was not complete for all the four years and all district hospitals in the study – thus resulting in inadequate number of hospitals for the MPI exercise. Furthermore, it was not possible to get complete and reliable data that could be used to unpack the causes of technical inefficiency using a second-stage Tobit regression analysis. This may also detract from the study's contribution to improving the way how hospitals operate.

## Competing interests

The author(s) declare that they have no competing interests.

## Authors' contributions

EZ designed the study, performed the analysis and drafted the report; TM participated in the collection of data, analysis and write-up of the report; WK and BT participated in data collection and report write-up; KS, CM and KM participated in the write-up and revision of the manuscript. All authors read and approved the final manuscript.
